# Changes in Training Quality and Workload Among Radiology Trainees Pre- and Post-COVID-19 Pandemic: A Systematic Review

**DOI:** 10.7759/cureus.117741

**Published:** 2026-10-10

**Authors:** Sammy Chan, Andrew Koo

**Affiliations:** 1 Radiology, Harrogate District Hospital, Harrogate, GBR; 2 Radiology, Harrogate and District NHS Foundation Trust, Harrogate, GBR

**Keywords:** covid-19, hybrid learning, quality, radiology education, radiology trainees, radiology training, simulation training, trainee workload, virtual reality

## Abstract

This review aimed to systematically review changes in radiology trainee training quality and workload before, during, and after the COVID-19 pandemic, assessing its impact on educational experiences, clinical and procedural exposure, workload demands, trainee wellbeing, and the adoption of adaptive educational strategies such as virtual learning and simulation-based training.

A systematic review was performed following the Preferred Reporting Items for Systematic Reviews and Meta-Analyses (PRISMA) guidelines. Electronic databases, including PubMed, Elsevier ScienceDirect and Web of Science, were searched for studies evaluating the impact of the COVID-19 pandemic on radiology trainee workload, education, and training quality. Supplementary searches of Google Scholar and relevant professional organisation websites, including the Royal College of Radiologists, were conducted, alongside targeted searches of *Clinical Radiology* and other relevant radiology publications. Although studies from multiple countries were included, findings were interpreted in the context of the UK radiology training curriculum and changes to radiology training and service provision during the COVID-19 pandemic. Eligible studies were analysed using narrative synthesis.

The COVID-19 pandemic significantly affected radiology trainee workload and training quality across multiple domains. Common findings included reduced clinical exposure, decreased reporting opportunities, loss of face-to-face teaching, reduced procedural experience, and concerns regarding competency attainment. Imaging volumes declined substantially because of the postponement of elective imaging and outpatient services, resulting in reduced exposure to radiographs, computed tomography (CT), magnetic resonance imaging (MRI), ultrasound, and interventional radiology procedures. Training progression was further affected by examination delays, rota disruptions, redeployment, and reduced consultant supervision. Increased psychological burden, including stress, anxiety, and burnout, was also reported across multiple studies. Despite these challenges, radiology training programmes demonstrated adaptability through the rapid adoption of virtual learning, remote reporting, simulation-based teaching, and digital educational platforms, improving accessibility and flexibility. These adaptations contributed to the development of increasingly hybrid training models combining virtual and face-to-face learning.

The COVID-19 pandemic substantially disrupted radiology trainee workload, training quality, clinical exposure, and procedural experience. However, it also accelerated the adoption of virtual learning, digital platforms, remote working, and simulation-based education. The post-pandemic period has therefore seen continued integration of these approaches alongside traditional apprenticeship-based training, with potential implications for the future delivery of radiology education and training.

## Introduction and background

The World Health Organisation (WHO) was officially informed of a cluster of pneumonia cases in Wuhan, China, on 31 December 2019. The causative pathogen was subsequently identified as severe acute respiratory syndrome coronavirus 2 (SARS-CoV-2), and on 11 March 2020, the WHO characterised coronavirus disease 2019 (COVID-19) as a global pandemic. By 31 December 2020, the global COVID-19-associated death toll had exceeded 1.8 million [1]. The pandemic disrupted healthcare systems worldwide, placing considerable strain on healthcare services and staff, including those within the National Health Service (NHS). Although this review includes studies from multiple countries, its findings are interpreted in the context of the UK radiology training curriculum and changes to radiology training and service provision during the pandemic.

The pandemic necessitated rapid service reconfiguration, staff redeployment, and implementation of social distancing measures, resulting in substantial changes to routine clinical practice. As healthcare systems prioritised urgent service delivery, many educational and specialty training opportunities were significantly affected [2].

Radiology training

Radiology training is a competency-based programme requiring trainees to develop expertise in interpreting a wide range of diagnostic imaging modalities, including radiographs (X-rays), ultrasound, computed tomography (CT), magnetic resonance imaging (MRI), positron emission tomography (PET), fluoroscopy, and nuclear medicine. Radiologists play a central role in modern healthcare through their contribution to the diagnosis, management, and monitoring of a wide range of medical conditions [3].

Radiological practice requires accurate and timely image interpretation, with imaging findings integrated with the clinical information provided by the referring clinician. Radiology reports can directly influence treatment decisions, surgical planning, and ongoing patient management. Trainees must therefore develop knowledge of anatomy, pathology, imaging physics, and disease processes alongside the ability to determine the clinical significance of imaging findings, recognise incidental abnormalities, prioritise urgent or life-threatening diagnoses, and communicate effectively with referring clinical teams [3,4].

Radiology training quality and workload prior to COVID-19

Before the COVID-19 pandemic, radiology training combined supervised clinical practice, formal teaching, and progressively greater responsibility for image interpretation and reporting. Trainees developed diagnostic accuracy, clinical reasoning, and communication skills through exposure to a range of imaging cases [5]. Consultant-supervised reporting sessions provided feedback on image interpretation and opportunities to develop diagnostic confidence and judgement [4].

Training also incorporated multidisciplinary team (MDT) meetings, protected teaching, and supervised practical experience. MDT meetings enabled trainees to relate imaging findings to clinical decisions, while hands-on ultrasound and image-guided procedures supported the development of technical skills, particularly for those pursuing interventional radiology [4]. These educational opportunities took place alongside service commitments in departments already facing increasing imaging demand and workforce pressures [6].

The pandemic disrupted this established training model through reduced elective imaging and outpatient activity, altered case volumes, restrictions on face-to-face reporting, and changes to service delivery, with potential consequences for trainee workload, supervision, procedural exposure, and educational opportunities [7,8]. However, the extent to which radiology training quality and workload changed across the pre-pandemic, pandemic, and post-pandemic periods remains unclear when evidence from different healthcare systems is considered collectively. The objective of this systematic review is therefore to compare changes in radiology trainee workload and training quality before, during, and after the COVID-19 pandemic, with particular focus on clinical and reporting exposure, procedural experience, supervision, educational opportunities, trainee wellbeing, and adaptations in training delivery. Findings from international studies are synthesised and considered in relation to the UK radiology training context.

## Review

Methods

This systematic review was conducted in accordance with the Preferred Reporting Items for Systematic Reviews and Meta-Analyses (PRISMA) guidelines to ensure methodological transparency and reproducibility [9]. The review question and search strategy were developed using the Population, Intervention, Comparison, Outcome (PICO) framework recommended by the Cochrane Handbook for Systematic Reviews of interventions [10]. The PICO elements were defined as follows: Population: radiology trainees, including residents, registrars, and fellows undertaking diagnostic or interventional radiology training programmes; Intervention: the COVID-19 pandemic and its associated effects on radiology training and clinical practice; Comparison: radiology training before the COVID-19 pandemic compared with training during and after the COVID-19 pandemic; Outcome: changes in trainee workload, quality of training, educational experience, reporting exposure, procedural exposure, examination and assessment opportunities, virtual or hybrid learning, wellbeing, burnout. 

Search Strategy

A literature search was conducted in June 2026 in accordance with the PRISMA 2020 guidelines. Electronic searches were performed in PubMed, Elsevier ScienceDirect, and Web of Science to identify studies evaluating the changes of the COVID-19 pandemic on radiology trainee workload, education, and training quality. Searches were limited to articles published between January 2020 and June 2026. Only English-language publications were included. 

The PubMed search combined Medical Subject Headings (MeSH) with free-text keywords using Boolean operators (AND/OR) to maximise search sensitivity. The search strategy included the following terms: ("Radiology” OR radiology) AND (resident OR trainee OR education OR training) AND (COVID-19 OR SARS-CoV-2 OR pandemic) AND (workload OR quality OR wellbeing OR burnout OR simulation OR "virtual learning"). The PubMed search was restricted to studies involving human participants.

ScienceDirect search used the following free-text search strategy: ("radiology resident" OR "radiology trainee" OR "radiology registrar") AND (COVID-19 OR pandemic) AND (training OR education OR workload). The ScienceDirect search was restricted to research articles and review articles. 

Web of Science search also used the following free-text search strategy: ("radiology resident" OR "radiology trainee" OR "radiology registrar") AND (COVID-19 OR pandemic) AND (training OR education OR workload). Web of Science search was restricted to articles as the document type. 

To maximise retrieval of relevant studies, supplementary searches were performed using Google Scholar with the keywords: radiology education, radiology training, and COVID-19. Additional manual searches were conducted by reviewing the Royal College of Radiologists website, screening articles published in Clinical Radiology, and examining the reference lists of all included studies to identify further eligible publications. Study screening and selection were performed by a single reviewer using predefined eligibility criteria. The absence of independent screening by a second reviewer may have increased the risk of missed eligible studies and selection bias.

Eligibility

Studies were eligible for inclusion if they investigated radiology trainees, including residents, registrars, or fellows, and evaluated the impact COVID-19 had on radiology training, workload, educational quality, reporting experience, procedural exposure, simulation-based education, virtual learning, wellbeing, or burnout. Original research studies published in English between January 2020 and June 2026 were eligible for inclusion if they reported outcomes relating to radiology trainees’ workload, education, or training quality during or following the COVID-19 pandemic. Relevant systematic reviews were examined to identify additional primary studies and provide background context, but their findings were not treated as independent study data in the synthesis. This approach avoided counting evidence from the same primary studies twice.

Studies were excluded if they did not include radiology trainees or reported outcomes exclusively for consultant radiologists or other healthcare professionals without trainee-specific data. Conference abstracts, editorials, letters, commentaries, case reports, and non-peer-reviewed publications were excluded. Studies published in languages other than English or those that did not report outcomes relevant to radiology training during or following the COVID-19 pandemic were also excluded.

Study Selection

Records identified through electronic database searches and supplementary searches were imported into Zotero reference management software, where duplicate records were identified and removed. Titles and abstracts were screened against the predefined eligibility criteria. Potentially eligible studies subsequently underwent full-text review to determine final inclusion. Reasons for exclusion at the full-text screening stage were recorded, and the overall study selection process is presented in the Preferred Reporting Items for Systematic Reviews and Meta-Analyses (PRISMA) flow diagram (Figure 1).

**Figure 1:**
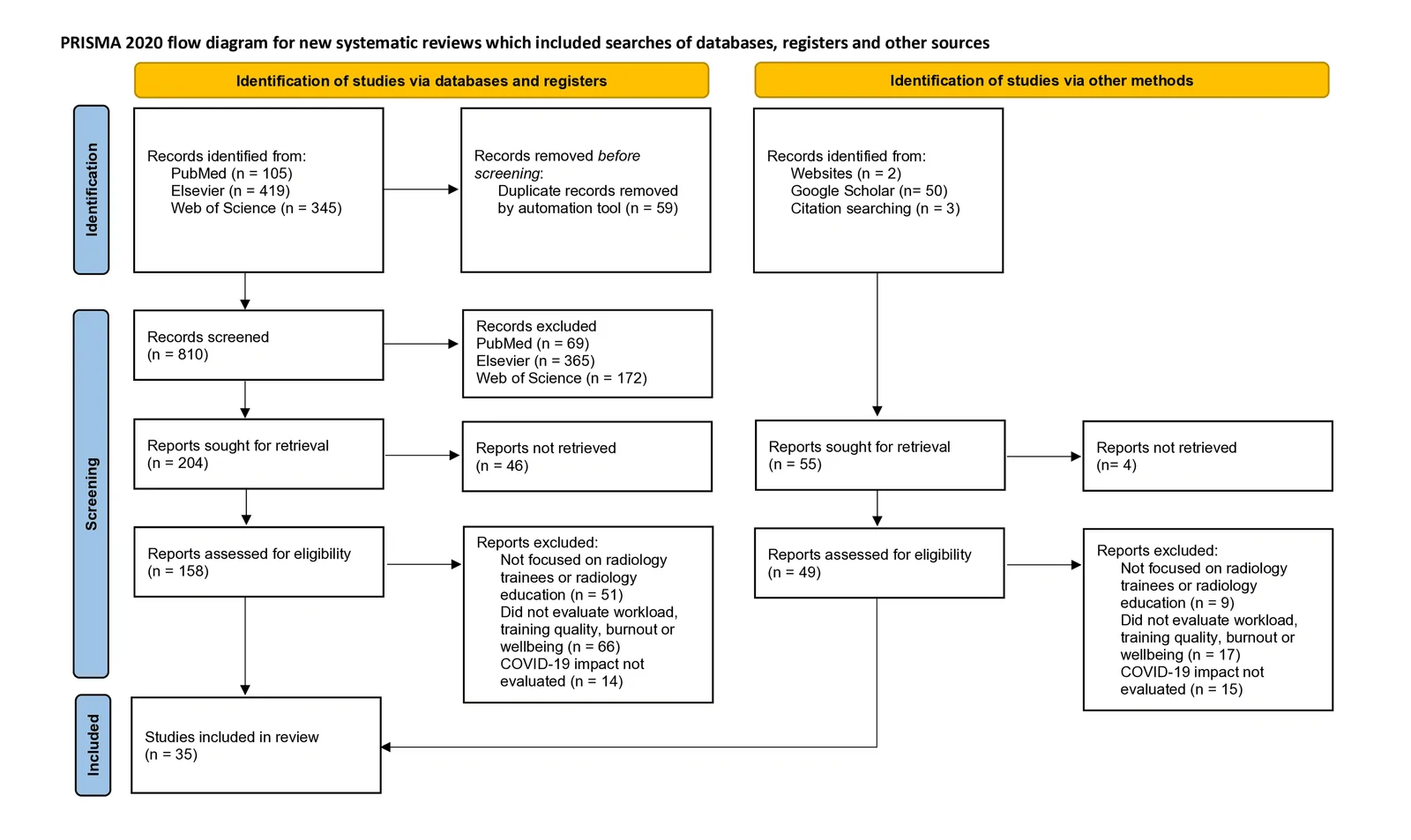
PRISMA 2020 flow diagram illustrating the identification, screening, eligibility assessment, and final inclusion of studies in the systematic review. PRISMA: Preferred Reporting Items for Systematic Reviews and Meta-Analyses

Data Extraction

Data were extracted using a standardised data extraction form. Extracted variables included first author, year of publication, country, study design, participant characteristics, sample size, radiology subspecialty where available, educational interventions, workload measures, educational outcomes, and findings relating to the impact of COVID-19 on radiology training. No formal quality or risk-of-bias assessment of the included studies was performed.

Data Synthesis 

Substantial heterogeneity was anticipated among the included studies with respect to study design, participant populations, outcome measures, and reporting methods. Consequently, quantitative pooling and meta-analysis were considered inappropriate. Findings were therefore synthesised using a narrative synthesis approach.

Studies were grouped according to recurring themes identified across the literature, including changes in training quality, trainee workload and on-call responsibilities, reporting exposure and clinical experience, procedural and interventional radiology exposure, reductions in face-to-face teaching, examination and competency concerns, the transition to virtual learning, simulation-based education, trainee wellbeing and burnout, and overall perceptions of radiology training during and following the COVID-19 pandemic.

Results

Study Characteristics

Following the study selection process, 35 studies met the predefined eligibility criteria and contributed to the narrative synthesis (Figure 1). The studies encompassed multiple countries and varied in population, sample size, methodology, and outcomes assessed, including trainee education, training quality, workload, clinical and procedural exposure, and wellbeing. Following review of all included studies, 12 were selected for Table 1 based on the comprehensiveness of their reporting of changes in trainee workload and training quality. Their characteristics and principal findings are summarised in Table 1. Although all 35 studies contributed to the synthesis, selection for the table involved subjective judgement and may have disproportionately highlighted the findings of these 12 studies.

**Table 1:** Evaluation of the impact of pre- and post-COVID-19 pandemic on radiology trainee education, training quality and workload.

Ref	Country	Institute	Study population (%)	Time of study	Survey tools	Findings
McDonnell et al. [11], 2022	UK, Australia	Department of Diagnostic Imaging, St James’s Street, Dublin, Ireland Radiology Department, Austin Hospital, Heidelberg, Australia Radiology Department, Mater Misericordia University Hospital, Dublin, Ireland	Radiology trainees (n=124)	Two weeks from 07/03/2021 -22/03/2021	Online survey	Unique representative of the entire national postgraduate training programme. Response rate of 51.6% from 124 radiology trainees. The experience of Irish trainees was similar to UK trainees, with the majority feeling that departmental workload decreased or remained the same and received no formal departmental teaching on reporting COVID-19 imaging. Growth of online education resources. Decreased consultant-led teaching during the pandemic. Overall better experience completing the FRCR exam in online format.
Pervez et al. [12], 2020	UK	Guy’s and St Thomas’s Hospital Radiology Department	Not applicable (discussion focuses on UK radiology trainees and training programmes)	COVID-19 pandemic, primarily 2020	Narrative	COVID-19 significantly disrupted radiology training through trainee redeployment, cancellation of FRCR examinations, reduced reporting opportunities, loss of procedural exposure (ultrasound, fluoroscopy, interventional radiology), reduced face-to-face teaching and feedback, and concerns regarding delayed progression and workforce shortages. The authors highlighted virtual learning platforms, video conferencing, online teaching files, and on-demand digital learning resources as potential solutions.
Veerasuri et al. [7], 2020	UK	Department of Radiology, Royal United Hospital	Radiology trainees (n=29)	Two weeks from the beginning of May	Online questionnaire	Radiology workload had significantly decreased during the COVID-19 pandemic significantly impacting radiology trainee reporting and experience. Overall trainee’s wellbeing was significantly negatively impacted. Response rate of 62%
Chong et al. [13], 2020	US	Department of Radiology, University of California San Diego	No formal participant cohort.	NA	Descriptive review	Radiology trainee workload decreased overall, particularly due to reduced imaging volumes, cancellation of elective services, and postponement of procedural rotations. Clinical responsibilities changed, with some residents redeployed outside radiology and others alternating between clinical work and home-based learning. Training quality was negatively affected by reduced reporting opportunities and procedural exposure. Virtual education expanded rapidly, partially mitigating the reduction in clinical learning opportunities.
McRoy et al. [14], 2020	US	Department of Diagnostic Radiology and Nuclear Medicine, University of Maryland School of Medicine,	Radiology trainees (n=16)	15^th^ Match to 15^th^ May 2020	Novel cloud-based Distance Learning Workstation	This model improved trainee’s confidence and knowledge during their on calls.
Odedra et al. [15], 2020	Canada	Canadian Association of Radiologists	Resident members of the Canadian Association of Radiologists (n=96)	1^st^ May – 15 May 2020	Online questionnaire	The pandemic significantly impacted radiology residents’ program and the experience. Greater disruption to daytime schedules and case volumes. Moderate disruption to teaching rounds. Virtual fellowship interviews were introduced. Internal and external assessments were severely affected. Psychological wellbeing of trainees was adversely affected.
Hoegger et al. [16], 2021	North America	86 institutions	Radiology chief residents (n=140)	20^th^ March to 15^th^ May 2020	Online questionnaire	59% reported increased stress. 93% reported fewer residents on clinical service.
Alhasan et al. [17], 2021	Saudi Arabia	National Survey	Radiology residents (n=109)	2019-2020 academic year	Online questionnaire	The majority of radiology residents reported they were negatively impacted by the pandemic on their education and clinical activities, and personal wellbeing.
Coppola et al. [18], 2021	Italy	National Survey	Members of the Italian Society of Medical and Interventional Radiology (n=2150)	2020	Online questionnaire	Members reported working and personal life were significantly impacted by the pandemic.
European Society of Radiologists (ESR) [3], 2020	34 European Countries	European Society of Radiology (ESR) member institution	Radiologists in training (n=249)	2020	Online survey	52.2% received no COVID-19-specific training; 78.7% were not redeployed; only 46.2% could work from home; e-learning available in 43% of departments
Fossey et al. [19], 2021	UK	Royal College of Radiologists, London, UK	Across 37 radiology training programmes across the UK	2021	National Survey	Ninety-five percent (38 of 40) of representatives completed the questionnaire 76% of trainees were redeployed to wards. Overall increase in attendance, volume, and quality of teaching and training nationally due to improved accessibility via remote learning methods
Patel et al. [20], 2021	Canada	National Survey	Interventional radiologists (n=142)	5^th^ May – 28^th^May, 2020	Online questionnaire	88% of Canadian departments reported overall decrease in elective IR services. 49.3% of participants reported a decrease demand for acute IR procedures. 73% of programs noted a drop in trainee case volumes by at least 25%

Impact on Training Quality

Studies reported disrupted subspecialty rotations, reduced reporting and procedural opportunities, decreased supervision and teaching, and interruptions to examinations and assessments [6-8,11,13]. Majumder et al. described reduced clinical exposure, case volumes, and trainee participation following the postponement of non-urgent imaging and elective procedures and restrictions on departmental attendance [8].

In a national Irish survey, 64 of 124 radiology trainees responded (51.6%). Of respondents, 54.7% reported a negative impact on overall training, while 32.8% considered it too early to assess the consequences. Disruption to subspecialty rotations, including neuroradiology and paediatric radiology, was reported by 77% [11].

Reduced Clinical Exposure 

Studies reported reduced imaging activity following the postponement of elective and non-urgent investigations [13,21,22]. Staffing changes included reduced on-site attendance, remote working, and reserve teams [13,21]. In North America, Hoegger et al. reported that 93% of surveyed programmes had fewer residents physically present on clinical services [16].

In the UK, Veerasuri et al. reported reductions in trainee reporting activity across several imaging modalities [7]. The proportion reporting more than 50 radiographs per week fell from 48% before the pandemic to 3% during it, while the proportion reporting fewer than 10 increased from 0% to 38%. For acute CT, the proportion reporting more than 30 examinations per week fell from 41% to 7%, while the proportion reporting fewer than 20 increased from 34% to 76% [7]. Pervez et al. also described reduced reporting and procedural opportunities in UK radiology training [12].

Participation in outpatient CT reporting decreased from more than 80% to approximately 50%. Among trainees competent in ultrasound, 64% performed 5-15 examinations per week before the pandemic, whereas 80% performed fewer than five during it. Reduced MRI exposure and interventional radiology procedural experience were reported by 73% and 85% of trainees, respectively [7].

Reduced Face-to-Face Teaching

During the COVID-19 pandemic, studies reported reductions in consultant-led teaching, workstation-based supervision, and in-person case discussion. McDonnell et al. described reduced consultant-led teaching and interaction with supervising consultants among Irish radiology trainees [11]. Pervez et al. reported reduced face-to-face teaching, feedback, and supervised clinical learning in UK radiology training [12].

Disruption also extended to scheduled educational activities. Odedra et al. reported disruption to teaching rounds among Canadian radiology residents [15]. These reports described changes to both formal teaching provision and routine trainee-consultant interaction.

Impact on Workload and Changes to On-Call Responsibilities

Changes in trainee workload varied between institutions. Among UK trainees surveyed by Veerasuri et al., 37.5% reported reduced overall workload, while 21.9% reported an increase [7]. Most trainees (61%) reported no increase in on-call workload, whereas 38.6% reported greater on-call demands and 15.6% reported increased on-call frequency. Staff absence due to COVID-19 infection or isolation contributed to these changes; 23.5% of trainees also lost more than seven days of training because of illness or mandatory isolation [7].

Trainee rotas were restructured through alternating on-site and off-site schedules, remote working, and separate staffing teams [23]. Alongside reduced elective activity, studies described a shift towards urgent and acute imaging and increased emphasis on maintaining clinical services [8].

Wellbeing

Studies reported increased stress, infection-related anxiety, isolation, and concerns about training progression among radiology trainees [16-18]. Hoegger et al. found that 59% of surveyed radiology chief residents reported increased stress [16]. In Saudi Arabia, Alhasan et al. reported that 80% of redeployed residents experienced a negative effect on wellbeing, with 38% describing the impact as severe [17].

In the Saudi survey, concerns included infecting family members (76.1%), contracting COVID-19 (58.7%), reduced clinical exposure (53.2%), and isolation (49.5%). Regarding psychological support, 51.4% were unaware of available mental health services, while 23.9% reported no access [17].

In the survey of 2,150 Italian radiology society members by Coppola et al., more than half of the trainee respondents reported increased emotional stress at work, alongside sleep disturbance and psychological exhaustion. More than 40% expressed moderate-to-severe concern about the long-term consequences of the pandemic for their professional development [18]. Other reports described examination delays, childcare responsibilities, reduced colleague interaction, and difficulties with remote learning, alongside variable psychological support provision [17,21].

Examination and Competency Concerns

Pervez et al. described cancellation of Fellowship of the Royal College of Radiologists (FRCR) examinations, interrupted rotations, and reduced reporting and procedural opportunities in UK radiology training [12]. Odedra et al. also reported substantial disruption to internal and external assessments among Canadian radiology residents [15].

Following the introduction of online examinations, McDonnell et al. reported that 87.5% of trainees who completed examinations remotely considered the experience similar to or better than in-person assessment [11]. Radiology residency and fellowship interviews also moved to virtual formats. Reported advantages included reduced travel requirements, financial costs, and time away from clinical duties [24]. Limitations included fewer opportunities to assess departmental culture and training environments directly, alongside concerns about equitable access to reliable internet connectivity and appropriate technological resources [24].

Transition to Online Learning

Radiology training programmes adopted virtual lectures, tutorials, video conferencing, and digital teaching resources during the pandemic [19]. McDonnell et al. reported growth in online educational resources and increased use of Zoom and Microsoft Teams for teaching [11]. Online delivery also enabled participation in teaching outside trainees’ local institutions [25]. Fossey et al. described widespread adoption of remote teaching across UK radiology training programmes [19].

Proposed approaches to support engagement included audience-response systems, live polling, and interactive case-based discussion [23]. Reports also described the potential for continued use of virtual and hybrid teaching beyond the acute pandemic response [19,23].

Benefits of Virtual Education

Upadhyay et al. described the suitability of digital imaging for web-based teaching, including remote case review and discussion [26]. Odedra et al. reported widespread use of online educational platforms among Canadian radiology residents, with Zoom frequently used and favourable trainee experiences of remote teaching [15].

Salajegheh et al. described web-based assessment, computer-assisted learning, mobile technologies, and other digital teaching tools [27]. Chytas et al. reported benefits of virtual reality (VR) for learning radiological anatomy and understanding three-dimensional anatomical relationships [28]. Technology-enhanced and simulation-based approaches were also explored as alternatives during restrictions on clinical training [29].

Limitations of Virtual Education and Interventional Radiology Exposure

Reports identified limitations in delivering hands-on ultrasound, fluoroscopy, and interventional radiology training remotely [12,20]. Veerasuri et al. reported reduced interventional radiology procedural exposure among 85% of trainees [7]. In Canada, Patel et al. found that 88% of departments reported reduced elective interventional activity, while 73% of programmes reported decreases in trainee case volumes of at least 25% [20].

Tay et al. discussed simulation as a potential means of supporting procedural learning during restricted clinical exposure [30]. For diagnostic training, McRoy et al. described a cloud-based platform incorporating a DICOM viewer for independent case interpretation, on-call preparation, and peer discussion [14].

Gaetke-Udager et al. reported that trainees valued quieter learning environments, flexibility, and opportunities to revisit teaching material. However, reduced interactivity and engagement were also reported [31].

Discussion 

This systematic review identified disruption to radiology trainees’ clinical exposure, workload, procedural experience, teaching, examinations, and wellbeing during the COVID-19 pandemic. Alongside these changes, the literature described an expansion of virtual education and alternative approaches to training delivery [32]. Maintaining formal teaching through digital platforms did not necessarily restore supervised reporting or procedural exposure, highlighting the distinction between continuity of teaching and continuity of clinical training. However, the predominantly descriptive and self-reported evidence limits conclusions about the extent of educational impairment and the effectiveness of these adaptations.

Clinical Exposure, Supervision and Workload

The findings suggest that workload and educational opportunity did not change in parallel. Reduced elective imaging lowered routine case volumes, while some trainees reported greater on-call demands and increased shift frequency [7]. Lower overall workload therefore cannot be interpreted as reduced service pressure or increased time for education. The concentration of activity around acute imaging may have preserved emergency reporting experience while reducing exposure to elective investigations and subspecialty cases [8]. This distinction is relevant when assessing training quality: the educational value of clinical activity depends on its breadth, supervision, and relevance to curriculum requirements, alongside the number of examinations reported. 

Disrupted subspecialty rotations added a further dimension to these changes, potentially narrowing opportunities to acquire experience across the curriculum. In the Irish survey, some trainees reported a negative impact on overall training, while others considered it too early to assess the consequences [11]. These responses highlight the difficulty of determining longer-term educational effects during an evolving disruption. Perceived training impairment should therefore be distinguished from demonstrated competency deficits, while recognising that missed rotations may require targeted opportunities to recover subspecialty exposure.

Reduced departmental attendance introduced a further distinction between access to cases and access to supervision. Even where reporting opportunities remained available, separation from supervising consultants may have changed the feedback accompanying those cases. Supervised reporting and discussion allow trainees to examine diagnostic uncertainty, understand discrepancies, and develop independent judgement [23,33]. Consequently, case volume alone may inadequately describe the educational effects of service reconfiguration. Trainee-trainer relationships additionally support mentorship and professional development [34]. Reporting volumes and scheduled teaching attendance may therefore capture only part of the educational experience affected by service reconfiguration.

The reductions across diagnostic and procedural modalities suggest that disruption extended beyond individual rotations [7,12,20]. However, the consequences are likely to depend on training stage, previous experience, and the duration of interruption. A temporary reduction in exposure may have different implications for a junior trainee acquiring basic skills and a senior trainee consolidating subspecialty or procedural competence. The available findings do not establish these differences, but they highlight the importance of considering individual training needs when interpreting aggregate survey results.

Although the studies were conducted across different healthcare systems, their findings are relevant to the UK training context because they concern reporting experience, subspecialty exposure, supervision, and procedural practice. However, differences in curricula, redeployment arrangements, and service organisation limit direct transfer of international findings to UK programmes. The review therefore identifies areas requiring attention rather than establishing a uniform effect on UK training.

Virtual Education and Procedural Training

Virtual education enabled lectures, recorded sessions, and digital case teaching to continue during restrictions on departmental attendance [11,19,26]. Teaching delivered beyond local institutions also broadened access to educational resources [25]. These adaptations addressed access to formal teaching, although they could not directly resolve every loss associated with reduced clinical activity.

The educational contribution of virtual learning also depends on how it is delivered. Recorded resources offer flexibility and repeated access, whereas live case discussions provide opportunities for questions and feedback. Reduced interactivity and engagement reported by trainees indicate that access to teaching does not guarantee active participation [31]. Satisfaction, attendance, knowledge acquisition, and clinical performance represent different outcomes; favourable findings in one should not be taken as evidence of improvement in the others. These distinctions are particularly relevant in departments facing increasing imaging demand and workforce pressures, where service commitments may constrain protected teaching and access to specialist educators [35,36]. Shared virtual teaching may broaden educational access, but its contribution should be evaluated alongside opportunities for supervised clinical learning.

Procedural training illustrates the limits of transferring education online. Knowledge of indications, anatomy, and procedural steps can be taught remotely, but opportunities to perform procedures under supervision depend on clinical activity. Reductions in interventional case volumes therefore represent a gap that increased lecture provision could not directly resolve [7,20]. Simulation may supplement restricted exposure [30], while remote diagnostic platforms can support case interpretation and on-call preparation [14]. Their value should be assessed against the particular skills they aim to develop, rather than assuming that all digital approaches provide equivalent substitutes for clinical experience.

Assessment, Recruitment, and Wellbeing

Examination cancellations and disrupted assessments affected the processes through which trainees demonstrate progression, alongside interruptions to learning opportunities [12,15]. These are related but distinct problems: delayed assessment may postpone recognition of competence, whereas reduced clinical exposure may affect its acquisition. The review cannot determine their relative contributions to longer-term progression. Similarly, favourable experiences of online examinations support their acceptability, but do not establish equivalent assessment validity, reliability, or fairness [11]. Evaluating remote assessment therefore requires consideration of both trainee experience and its ability to assess the intended competencies.

Virtual recruitment reduced travel requirements and financial costs, while limiting opportunities to observe departments and assess programme suitability directly [24]. These advantages and limitations do not establish whether virtual interviews improved equitable access or affected subsequent satisfaction with training. Their evaluation should therefore consider both the interview experience and applicants’ ability to make informed choices, including access to reliable technology and meaningful interaction with prospective programmes.

The wellbeing findings suggest that distress was associated with both personal health concerns and uncertainty about training [16-18]. Reduced workload should consequently not be assumed to improve wellbeing: fewer clinical cases could coexist with infection-related anxiety, isolation, or concern about professional development. Predominantly cross-sectional, self-reported evidence limits conclusions about whether changes in training caused distress or whether difficulties persisted after educational activities resumed. Reported stress should also be distinguished from clinically diagnosed mental health conditions.

Reported gaps in psychological support also distinguish service availability from trainees’ awareness of and access to those services [17,21]. These findings do not establish whether available services were used, met trainees’ needs, or improved wellbeing. Support provision should therefore be evaluated through accessibility, uptake, and outcomes, alongside clear communication about assessments and progression. Maintaining teaching provision alone may not address the range of difficulties experienced during major service disruption.

Limitations of the Evidence and Review Methodology

Interpretation is limited by variation in study populations, healthcare settings, training stages, methods, and outcome measures. Much of the evidence comprised cross-sectional surveys, descriptive studies, and self-reported experiences, restricting conclusions about causality and objectively measured educational outcomes. Studies conducted during the early pandemic may inadequately represent subsequent recovery. Later publication dates do not necessarily indicate post-pandemic follow-up, as articles may report data collected during earlier phases. Consequently, the January 2020 to June 2026 publication window does not itself establish evidence of persistent deficits or recovery.

The review methodology also introduces limitations. Screening and selection were performed by a single reviewer, increasing the possibility of missed studies and inconsistent eligibility decisions. Searches included PubMed, ScienceDirect, and Web of Science, alongside supplementary searches, but the absence of other journal databases may have limited retrieval. Restriction to English-language publications may have excluded relevant evidence and reduced representation of some training settings. No formal quality or risk-of-bias assessment was performed, limiting the ability to evaluate methodological weaknesses and account for them in the synthesis.

All 35 studies contributed to the narrative synthesis, but selecting 12 for Table 1 involved subjective judgement about the comprehensiveness of reporting and may have disproportionately highlighted their findings. Including systematic reviews alongside original studies also creates potential overlap in the underlying evidence; findings reported through overlapping sources should not be interpreted as independent confirmation. These limitations reduce confidence in conclusions about the magnitude, duration, and generalisability of the reported changes.

Implications and Future Research

A blended approach combining supervised clinical experience with selected digital resources provides a potential framework for future training [37]. The findings support evaluating teaching formats according to the competencies they are intended to develop, while preserving opportunities for direct feedback and procedural practice. Simulation and virtual reality warrant focused evaluation as adjuncts to training, rather than assumptions that they can replace clinical exposure [28,30]. Shared digital case repositories and adaptive learning platforms could also be investigated, although their effectiveness was not established by this review.

Future research should use clearly defined data-collection periods to distinguish pre-pandemic training, pandemic disruption, and subsequent recovery. Longitudinal studies incorporating reporting performance, procedural competence, examination outcomes, progression, and wellbeing would help determine whether perceived disruption translated into lasting consequences. Comparative evaluation of face-to-face, virtual, and hybrid teaching should distinguish accessibility and satisfaction from sustained learning and clinical performance.

## Conclusions

The COVID-19 pandemic disrupted radiology training through reduced clinical and procedural exposure, interrupted supervision and assessments, variable workload and on-call demands, and concerns about trainee wellbeing. Virtual education helped maintain formal teaching and broaden access to resources, but did not fully compensate for reduced supervised clinical experience. The evidence primarily reflects trainee-reported disruption rather than demonstrated long-term competency deficits.

These findings support combining accessible digital teaching with supervised reporting, hands-on procedural training, and wellbeing support. However, limitations in the evidence and review methodology restrict conclusions about lasting effects and the effectiveness of educational adaptations. Longitudinal evaluation is needed to assess training recovery, competency attainment, and the sustained value of hybrid education.
